# Effect of Whole-Body Vibration Training on Selected Intrinsic Risk Factors in Women Aged 60+ at Fall Risk: A Randomized Controlled Trial

**DOI:** 10.3390/ijerph192417066

**Published:** 2022-12-19

**Authors:** Agnieszka Nawrat-Szołtysik, Marta Sieradzka, Marta Nowacka-Chmielewska, Laura Piejko, Julia Duda, Anna Brachman, Anna Polak

**Affiliations:** 1 Institute of Physiotherapy and Health Sciences, Jerzy Kukuczka Academy of Physical Education, 40-065 Katowice, Poland; 2 Doctoral School, Jerzy Kukuczka Academy of Physical Education, 40-065 Katowice, Poland; 3 Laboratory of Molecular Biology, Institute of Physiotherapy and Health Sciences, Jerzy Kukuczka Academy of Physical Education, 40-065 Katowice, Poland; 4 Institute of Sport Sciences, Jerzy Kukuczka Academy of Physical Education, 40-065 Katowice, Poland

**Keywords:** IL-6, TUG, 30SCST, 6MWT, WBVT, FES-I, body imbalance, risk of falling, geriatric rehabilitation

## Abstract

The aim of the study was to determine whether Whole Body Vibration Training (WBVT) affects intrinsic risk factors for falls in women aged 60+ at fall risk. Design: Randomized controlled clinical trial. Blinding was applied to the persons in charge of evaluating the intervention’s clinical results and statistical analysis. Methods: Forty-two women over 60 years old were randomly assigned to an experimental group (EG—12-week WBVT; n = 22) and a control group (CG—no additional physical activities; n = 20). Fear of falling was measured by the FES-I questionnaire, gait and dynamic balance using the Time-Up and Go test (TUG), aerobic endurance with the 6-Minute Walk Test (6MWT), and the functional strength of the lower body muscles with the 30-s Chair Stand Test (30SCST) at baseline and post-intervention. Additionally assayed were participants’ blood concentrations of interleukin-6 (IL-6). Results: The 12-week WBVT improves gait and balance (TUG, *p* = 0.009), exercise tolerance (6MWT, *p* = 0.001), and functional strength (30SCST; *p* = 0.027) but does not reduce the intensity of fear of falling (FES-I, *p* = 0.655) and the IL-6 serum concentration (*p* = 0.377). Conclusions: WBVT affects selected fall risk factors in women aged 60+ at fall risk.

## 1. Introduction

Adults’ risk of falling increases with age. Epidemiological data show that 18% of people under the age of 45, 25% of those aged 45–65 years, and 30% of those aged 65+ suffer falls each year [1]. Among 70-year-olds, the rate is as high as 53% [2].

The main intrinsic fall risk factors include gender (the incidence of falls is higher among women than among men) [3,4] low physical activity, mobility and functional impairments, fear of falling after a fall, decreased muscle strength, and gait and balance disorders [5]. An association has also been found between cognitive function impairment and increased fall risk [6,7].

Physical activity plays an important role in preventing falls [8], as it helps maintain muscle mass, improves motor coordination, and reduces the risk of balance disorders [9].

There is evidence that seniors’ risk of falling can also be reduced by Whole-Body Vibration Training (WBVT) [10,11,12] on a force plate oscillating with a frequency and amplitude forcing muscle activity similar to the tonic vibration reflex [13].

Randomized clinical trials (RCTs) show that a combination of physical exercises and WBVT can increase the strength of knee joints’ flexors [14] and extensors [14,15], the explosive power of the lower body muscles [14], and the extension speed of the knee [14] in seniors. WBVT also improves functional gait efficiency and speed by increasing bioelectrical activity of the plantar flexors responsible for the heel-stroke/toes-off phase [15,16], dynamic balance [14,17], and functional independence, and mitigates the consequences of falls [17]. Overall, the results of the cited studies confirm the ability of WBVT to make older adults less prone to intrinsic fall risks. However, as studies of this type are still few, more clinical research is needed to confirm their findings, especially that the experimental groups in the cited studies also received other treatments in addition to WBVT, i.e., balance exercises [16,17] and strength and endurance exercises [14,15,17], whose results were compared with controls.

Research has shown that older adults have elevated blood concentrations of pro-inflammatory factors, including interleukin 6 (IL-6) [5]. It is one of the main reasons for seniors’ greater sensitivity to pain, fatigue, concentration problems, and impaired postural balance, which altogether expose them to a higher risk of falling [7].

Given that, it seems important to determine whether WBVT can influence the concentration of pro-inflammatory cytokines in healthy seniors at increased risk of falling due to their age. The only clinical trial in this area known to the authors, conducted by Cristi et al., failed to find changes in IL-6 blood concentrations in older women after WBVT intervention. However, being obtained during a preliminary trial without a control group, the result should be interpreted with caution and needs to be assessed by other clinical trials [18].

Our study is the first randomized clinical trial with women aged 60+ and a control group, which assessed WBVT’s influence on functional gait efficiency, dynamic balance, fear of falling, and the functional strength of the lower body muscles, also examined its effect on their exercise tolerance and serum concentrations of IL-6.

### Aim

The trial aimed to determine whether and how whole-body vibration training influences selected intrinsic fall risk factors in women aged 60+ at increased risk of falling. To this end, answers to the following research questions were sought: (1) Does WBVT influence and how functional gait and dynamic balance, exercise tolerance, the functional strength of the lower body muscles, fear of falling, and the serum concentration of IL-6 in women aged 60+, and what is the influence? (2) Is the serum concentration of IL-6 in women aged 60+ correlated with functional gait efficiency and dynamic balance, exercise tolerance, and the functional strength of the lower body muscles?

The practical purpose of the study was to identify WBVT protocols that can be the most effective in protecting women aged 60+ from the intrinsic risk factors of falls.

## 2. Materials and Methods

### 2.1. Study Design

The study was conducted between 1 June and 31 October 2018, with 60 women who volunteered to participate in it.

It was designed as a prospective, randomized, controlled clinical trial to compare intrinsic fall risk factors between women aged 60+ who performed WVBT for 12 weeks and same-age controls whose physical activity was limited to daily routines. The trial’s registration number was: ISRCTN69374524.

### 2.2. Bioethical Approval

The study was approved by the Bioethics Commission at the J. Kukuczka Academy of Physical Education in Katowice, certificate number 1/2018, on 15 November 2018.

### 2.3. Study Inclusion and Exclusion Criteria

Patients’ eligibility for the study was determined by the same physician. The study enrolled women aged 60+ who resided in the same long-term care facility or lived in their own homes, after obtaining their informed consent. All participants had scores above 20 on the Barthel Scale measuring independence in daily activities. They could make logical verbal communication, had the Mini-Mental State Examination (MMSE) scores of ≥24 points, and could understand and follow physiotherapist’s instructions. Each woman had fallen at least once during the 12 months preceding the study and felt fear of falling again (scores of more than 16 points on the Falls Efficacy Scale International (FES-I)).

The exclusion criteria included contraindications for vibration therapy (acute inflammation conditions, osteoporosis, bone fractures or other injuries to the lower limbs or the backbone suffered within 12 months prior to the commencement of the study, ongoing therapy for malignant tumors, diabetes, acute and chronic vascular and lymphatic diseases, risk of internal or external bleeding), and disease-related balance and mobility problems [14,15,16,17].

### 2.4. Information for Patientsand Randomisation to Groups

The enrolled women were informed in writing about the plan and purpose of the study and that they could withdraw from it at their discretion without giving a reason. They were also assured in writing that the decision to withdraw would not affect their treatment.

The women were randomly allocated to two groups. In preparation for the study, 46 opaque envelopes and the same number of slips of paper marked with letters A (the control group (CG), 23 slips) or B (the experimental group (EG), 23 slips) were produced. The envelopes and the slips were given over to a person uninvolved in the study, who placed one slip in each of the envelopes, sealed them, and numbered them randomly from 1 to 46. The envelopes were then delivered to the principal investigator, who opened them one by one to assign each woman to a group according to the symbol.

For ethical reasons, all women assigned to the control group were offered participation in vibration training after the study.

### 2.5. Blinding

The physician examining patients’ eligibility for the study was unaware of and could not influence their future group allocation. Blinding was applied to the person in charge of functional tests, the investigator assessing the clinical results of the intervention, the laboratory technician assaying serum IL + 6 concentrations, and the statistician.

### 2.6. Intervention

In addition to normal physical activity that both groups performed over the 12 weeks of the study, the EG women participated in WBVT sessions. All sessions were held in the same physiotherapy center housed in the long-term care facility and were supervised by the same physiotherapist.

The WBVT protocol was designed based on clinical trials applying vibration training to people aged 60+ [14,15,17] and provided for the use of the Fitvibe 600 platform (Gymna Uniphy, NV; Limburg, Belgium). The EG attended two weekly WBVT sessions (Mondays and Thursdays) for 12 weeks. The frequency of vibrations was set at 20 Hz and the amplitude at 2 mm. A session lasted 10 min and involved five exercise cycles, with one cycle consisting of 1-min vibrations separated by a 1 min interval.

**Figure 1:**
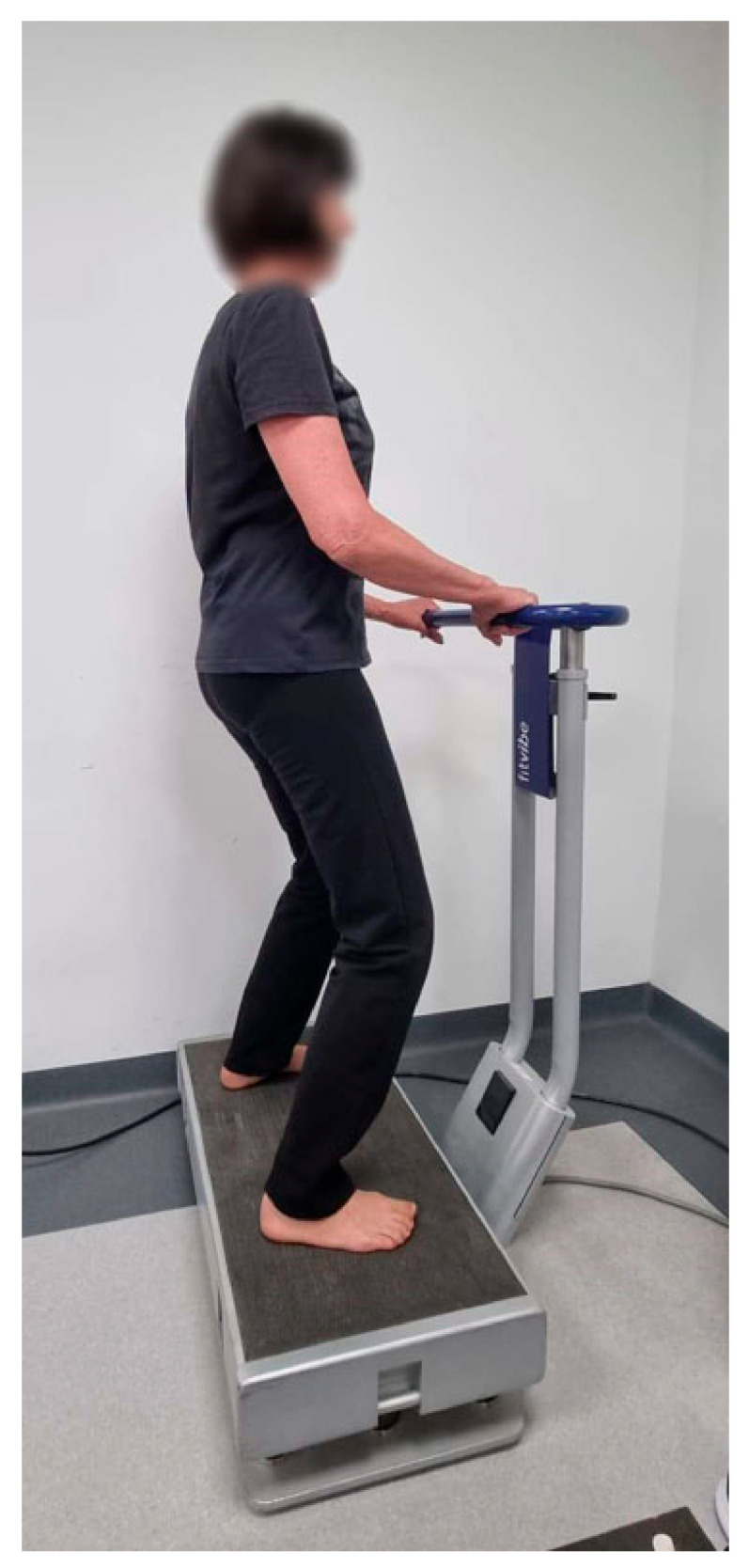
WBVT training in a semi-squat position.

The participants were briefed on how the platform worked before the intervention. They were asked to wear comfortable sports clothing for exercise sessions, which required them to stand barefoot on the force plate in a relaxed or semi-squat position with knees bent to 35° allowing isometric contractions of the muscles. While exercising, they held on to a handrail in front of them for safety (Figure 1).

### 2.7. Measures

Patients’ independence in performing daily activities and their cognitive abilities were examined before the study began.

Independence in activities of daily living was determined on a 100-point Barthel scale measuring items such as feeding, personal toileting, bathing, dressing, undressing, controlling bladder and bowel, walking on a flat surface, ascending and descending stairs, etc. A score of 0–20 indicates total dependency, 21–85 points indicate mild functional dependency, and a score of between 86 and 100 means that a person is entirely independent in daily activities [19].

Participants’ cognitive function (orientation to time and place, short-term memory (recall), attention/concentration, calculation, language, and constructional praxis) was assessed using the Mini-Mental State Examination (MMSE) questionnaire, which contains 12 self-completed questions and 8 tasks. The MMSE has a maximum score of 30 divided into five parts: 27–30—normal cognition, 24–26—mild cognitive impairment without dementia, 19–23—mild dementia, 11–18—moderate dementia, and 0–10—severe dementia [20].

Participants’ susceptibility to intrinsic risk factors of falls was assessed at baseline and immediately post-intervention using the Time-Up and Go test (TUG) of functional gait efficiency and dynamic balance, the 6-Minute Walk Test (6MWT) of exercise tolerance, and the 30-s Chair Stand Test (30sCST) of the functional strength of the lower body muscles. Fear of falling was evaluated on the Falls Efficacy Scale International (FES-I). During the tests, patients wore comfortable sports clothing and lace-up trainers.

The Time-Up and Go test measuring functional gait efficiency and dynamic balance required a patient to rise from a chair on the command ‘go, walk a distance of 3 m, turn, walk back, and sit on the chair again. The start and end lines were clearly marked out on the floor. Patients were given specific instructions on how to perform the test and were allowed two trials without taking their time before it began. Then, they performed two trials whose times were averaged and subjected to analysis [21].

The 6-min Walk Test allowing exercise tolerance to be assessed was conducted in a long hallway where patients could safely walk over a distance of 30 m. The start and turn-back lines were marked out on the floor 30 m apart by horizontal lines and road cones. Additionally, 10 segments of 3 m were marked out with transverse lines. The outcome of the test was the distance walked over 6 min [22].

In the 30-s Chair Stand Test measuring the functional strength of the lower body muscles, a patient sitting on a chair (with a 45 cm seat height, straight back, and without armrests) with hands crossed on the opposite shoulders, and feet positioned parallel at hip width was asked to rise to a full standing position and sit back down again as many times as they could during a measured time of 30 s. The test started on the physiotherapist’s command. The outcome of the test was the number of full standing and sitting cycles [23].

Patients’ fear of falling was assessed using the Falls Efficacy Scale International questionnaire that they completed themselves as per the physiotherapist’s instructions. The FES-I was created to measure the fear of falling during daily activities such as walking around the flat, sitting and standing from a chair, opening the door, and dressing an undressing. Each FES-I item is rated on a scale of 0 (no fear) to 10 (maximum fear). The total score of minimum 10 and maximum 100 points is calculated by adding up the scores for particular items [24].

The serum IL-6 concentrations were determined from 5 mL samples of venous blood, which were collected from the participants between 7 a.m. and 9 a.m. and assayed for IL-6 by the immunoenzymatic method (ELISA) (IL-6 kit by R&D Systems, Biotechene, D6050).

The participants were familiarized with the selected tests and measurements before the intervention. The interval between the end of the training period and the next measurements was a maximum of 3 days.

### 2.8. Primary Study Outcomes

The primary study outcomes included the levels of functional gait efficiency and dynamic balance (TUG: pre-study (TUG-0) and post-study (TUG-1) time (s)), exercise tolerance (6MWT: pre-study (6MTW-0) and post-study (6MTW-1) distance [m]), functional strength of the lower body muscles (30sCST: pre-study (30sCTS-0) and post-study (30sCTS-1) numbers of repetitions (no. of repetitions)), the intensity of fear of falling (FES-I: pre-study (FES-0) and post-study (FES-1) intensity of fear of falling (score)), and pre-study (IL6-0) and post-study (IL6-1) concentrations of IL-6 (pg/mL)).

The primary outcomes were compared between the EG and the CG by calculating percentage differences between baseline and week-12 values of particular variables using the following formulas:

(1) Percentage change in TUG times (%TUG):
  %TUG = (TUG-1 − TUG-0/TUG-0) × 100%,
  where %TUG—percentage change in TUG result
(2) Percentage change in 6MWT distances (%6MWT):
  %6MWT = (6MWT-1 − 6MWT-0/6MWT-0) × 100%,
  where %6MWT—percentage change in 6MWT distance
(3) Percentage change in the number of repetitions in the 30sCST (%30sCST):
  %30sCST = (30sCST-1 − 30sCST-0/30sCST-0) × 100%,
  where %30sCST—percentage change in the number of repetitions in the 30sCST
(4) Percentage change in the fear of falling (%FESI):
  %FESI = (FESI-1 − FESI-0/FESI-0) × 100%,
  where %FESI—percentage change in the intensity of fear of falling
(5) Percentage change in the serum IL-6 concentration
  %IL6 = (IL6-1 − IL6-0/IL6-0) × 100%,
  where %IL6—percentage change in the serum IL-6 concentration

### 2.9. Secondary Study Outcome

The secondary outcome of the study was correlations between the participants’ serum IL-6 concentrations and functional gait efficiency and dynamic balance (TUG results), exercise tolerance (6MWT results), functional strength of the lower body muscles (30sCST results), and intensity of fear of falling (FES-I scores) obtained for both groups before and after the intervention.

### 2.10. Statistical Analysis

Statistical analysis was performed in Statistica software (v. 12/2021, Stat Soft Polska, Sp. z o.o.). The level of significance for all tests was *p* ≤ 0.05.

The normality of distribution of variables characterizing the participants and the homogeneity of variance were tested by the Shapiro-Wilk test and Leven’s test, respectively. As distributions proved to be not normal and variances were not homogeneous, statistical analysis was conducted using non-parametric tests. To account for the fact that the values of skewness and kurtosis were smaller than 2.5 and the variables’ distributions were unimodal, means and standard deviations (the measures of location and dispersion, respectively) were calculated, in addition to medians and quartiles.

The baseline characteristics of patients in both groups were compared using the maximum likelihood chi-square (Chi2) test and the Mann–Whitney *U* test.

The baseline and post-intervention results of the functional tests (TUG, 6MWT, 30sCST) and FES-I scores were compared between the groups using the Wilcoxon test for paired samples. The same test was also used to make pre-post comparisons of IL-6 concentrations.

Percentage changes in the results of the functional tests (TUG, 6MWT, and 30sCST); FES-I scores; and the serum concentrations of IL-6 were compared between the groups with the Mann–Whitney *U* test.

Correlations between the serum Il-6 concentrations and the results of the functional tests (TUG, 6MWT, and 30sCST) were established using the Spearman correlation of ranks.

## 3. Results

**Figure 2:**
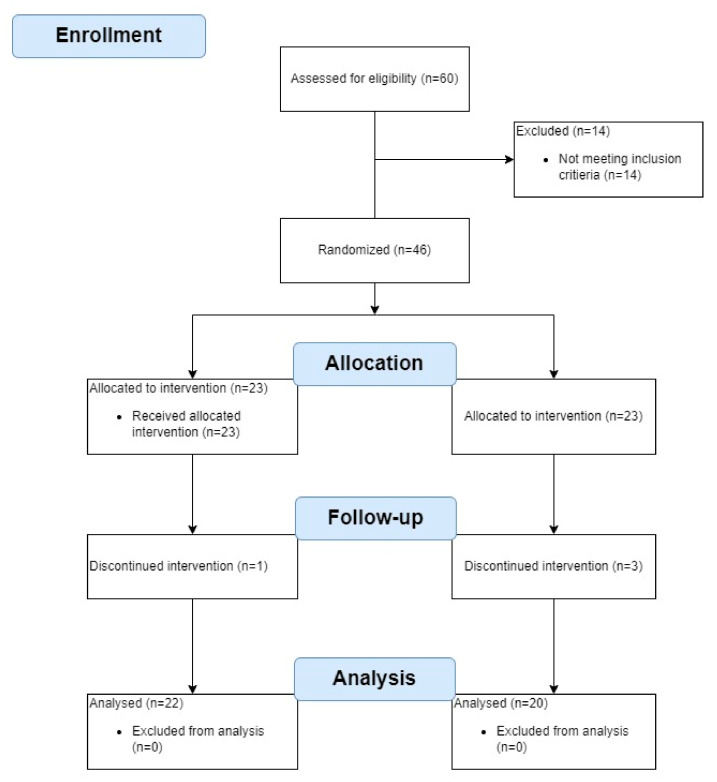
The research flowchart.

A total of 60 women volunteered to participate in the study. Fourteen women were disqualified as they did not meet the inclusion criteria (two had MMSE scores below 24, three scored 16 on the FES-I, two had been diagnosed with nervous system diseases, one had suffered a lower extremity fracture before, one had osteoporosis, and five had cardiovascular conditions). The other 46 women were randomly assigned to an experimental group (EG; n = 23) or a control group (GC; n = 23). Of those, four women (8.7%) did not complete the study: two (one in the EG and one in the CG) dropped out due to health problems unrelated to the study, and two (CG) withdrew for other reasons. Statistical analysis was performed on the results obtained for the remaining 42 women (22 in the EG and 20 in the CG). The research stages are presented in Figure 2.

### 3.1. Participants’ Basic Characteristics

The study participants were aged 60 to 85 years and had a body mass index of between 17.8 and 39.3 kg/m^2^. Seventeen women (40.48%) were the residents of a long-term care facility, and the other twenty-five (59.52%) lived in their own homes.

Participants’ baseline scores on the Barthel Scale ranged from 85 to 10, meaning that they were independent in daily activities and only occasionally needed assistance.

Participants’ MMSE scores ranged between 26 and 30, indicating normal cognitive function (27–30 pts) or only mild impairment without dementia (24–26 pts).

All women had fallen at least once during the 12 months preceding the study and felt a fear of falling. The fear was low in 19 (45.24%) of them (FES-I score of 17–19); in 14 women (33.33%), it was moderate (20–27); and 9 women (21.43%) were very concerned that they might fall again (20–27).

The TUG test performance times ranged from 5.37 to 14.3 s. Twenty on women (50%) completed the test in 10–14 s, which indicated a slight impairment of functional gait efficiency and dynamic balance, and two women (4.76%) needed slightly over 14 s to complete it, which pointed to a significant impairment of functional gait efficiency and dynamic balance involving a high risk of falling.

The number of repetitions performed by 10 patients (23.81%) in the 30sCSTwas below their age norm, indicating lower functional strength of the lower body muscles.

The distance walked by the patients in the 6MWT varied from 135 to 475 m. Based on their ages and individual results, it was determined that exercise tolerance was impaired moderately in 30 (71.43%) women and significantly in 2 women (4.76%).

Participants’ serum IL-6 concentrations were in the range of 1.34–12.30 pg/mL. While a reference range for this interleukin has not been developed, the values were low enough to practically preclude the presence of acute chronic conditions.

The study groups were not statistically significantly different at baseline in participants’ baseline characteristics (*p* > 0.05) (Table 1).

### 3.2. Primary Study Outcomes

The EG’s performance time on the TUG test decreased after WBVT by 17% (SD 0.29%) on average, but in the CG, it was longer by an average of 7% (SD 12%). The difference between the groups was statistically significant in favor of the EG (*p* = 0.009) (Table 2).

The EG also better performed on the 6MWT, by an average of 50% (SD 42%). The CG women walked a longer distance, too, but the increase was only 9% (SD 28%). The difference between the groups was statistically significant in favor of the experimental group again (*p* = 0.001) (Table 2).

The number of repetitions completed by the EG in the30sCST at week 12 was statistically significantly greater (*p* = 0.027) compared with the CG; it increased from baseline by an average of 19% (SD 33%), whereas, in the CG, it decreased by 5% (SD 15%) (Table 2).

The change in the intensity of fear of falling between baseline and week 12 was not statistically significantly different (*p* = 0.655) between the EG and CG. Both groups’ scores on the FES-I decreased by an average of 5% (SD 17) and 2% (SD 21), respectively (Table 2).

The serum IL-6 serum concentration in the EG decreased by an average of 2% (SD 34%), whereas in the CG, it decreased by 39% (SD 108%); the difference between the groups was not statistically significant (*p* = 0.377) (Table 2).

### 3.3. Secondary Study Outcome

Statistically significant correlations between the participants’ serum IL-6 concentrations and their results on the functional tests (TUG, 6MWT, and 30sCST) were not observed either at baseline or at week 12 (Table 3 and Table 4).

WBVT did not deteriorate patients’ health or have adverse effects that might require terminating the intervention. The participants only occasionally reported muscle fatigue after vibration exercise, which subsided by the next session.

## 4. Discussion

The results of our study show that vibration training (WBVT) can reduce intrinsic risk factors of falls in women who experience falls more often than men. The study is novel as the first randomized clinical trial, which assessed the effect of WBVT on intrinsic fall risk factors (including exercise tolerance and serum IL-6 concentrations) in women aged 60+ at an increased risk of falls.

### 4.1. Functional Tests

Twelve weeks of whole-body vibration training significantly reduced the time needed by the EG to perform the TUG test (*p* = 0.009) compared with the CG, meaning that WBVT had a positive effect on these participants’ functional gait efficiency and dynamic balance. Verschueren et al. [14] and Pollock et al. [17] also reported improved functional gait efficiency [17] and dynamic balance in women [14,17] and men aged 60+ [17] after WBVT.

The number of repetitions performed by the EG in the 30sCST increased significantly (*p* = 0.027) between baseline and week 12 compared with the CG, pointing to WBVT’s ability to increase the functional strength of the lower body muscles. Increased strength of the lower body muscles in women aged 60+ after vibration training was also reported by Roelants et al. [15] and Verschueren et al. [14]. Moreover, Roelants et al. [15], Ochi et al. [16], and Verschueren et al. [14] observed a statistically significant increase in the speed of knee extension [15] and bioelectrical activity of the triceps muscle of the calf engaged in the heel strike and toe off phase of gait in seniors participating in WBVT sessions [16].

The distance walked by the EG women in our study at week 12 was significantly longer (*p* = 0.001) than at baseline, by an average of 50% compared with the CG. Therefore, WBVT appears capable of improving exercise tolerance in women aged 60+. Our results are not directly comparable with the findings reported by the cited authors, who did not evaluate WBVT’s influence on exercise tolerance.

Roelants et al. [15] and Verschueren et al. [14], who used the same WBVT protocol, also found vibration training to improve dynamic balance and functional strength of the lower body muscles in women aged 60+. Participants in both studies performed 3 WBVT sessions per week for 24 weeks. The frequency and amplitude of vibrations were progressively increased from 35 to 40 Hz and 2.5 to 5 mm, respectively. The duration of exercise was extended from 3 min in week 1 to 30 min in week 24. One session consisted of several to 10–20 cycles of vibrations. The duration of vibrations was gradually increased from 30 to 60 s while the interval between them was reduced from 60 to 5 s [14,15].

Pollock et al. [17] reported improved dynamic balance in seniors who participated in 3 WBVT sessions over 8 weeks. The frequency and amplitude of vibrations applied during sessions were progressively increased from session to session from 15 to 30 Hz and from 2 to 8 mm, respectively. An exercise session comprised five cycles, with one cycle consisting of vibrations applied for 1 min and a 30-s interval [17].

Our study participants attended WBVT sessions for 12 weeks. The frequency and amplitude of vibrations (20 Hz and 2 mm, respectively) were kept constant throughout the intervention. One session included 5 cycles, each consisting of vibrations applied for 1 min and a 1-min interval.

Our and Roelants et al.’s results [15] are different from those published by Pollock et al. [17], Verschueren et al. [14], and Ochi et al. [16], who did not find improvements in the dynamic balance and maximum strength of the knee extensors in women aged 60+ after 12 weeks of WBVT [16]. The difference is difficult to explain. Our study’s participants were younger than those studied by Ochi’ et al. (69 ± 6 years vs. 80 ± 2.8 years), Verschueren et al. (64.6 ± 3.3 years) [14], and Roelants et al. (64.6 ± 0.7 years) [15]), but of comparable age with the women studied by Pollock et al. (80 ± 1.4 years) [17]. In our and all cited studies [14,15,17], the participants were healthy older adults with balance disorders, walking problems, and reduced muscle strength caused exclusively by older age and not concomitant diseases. A comparison of WBVT protocols shows that patients studied by Ochi et al. [16] performed one WBVT session daily and were treated with vibrations continually for 3 min. In our and the other cited studies [14,15,17], the participants received several cycles consisting of vibrations and intervals, and the total exercise time was longer than in Ochi et al. [16]. Whether these methodological differences could cause Ochi et al. [16] to obtain different results cannot be firmly answered without further clinical research. The other WBVT parameters selected by Ochi et al. [16] were comparable with those used by us and the other authors [14,15,17]. Ochi et al. [16] had their participants perform 3 WBVT sessions per week for 12 weeks and increased vibration frequency and amplitude from 10 to 21 Hz and 3 to 12 mm, respectively.

### 4.2. Fear of Falling

In our study, WBVT did not moderate participants’ fear of falling as measured on the FES-I scale. Pollock et al. [17] did not find changes in older women’s and men’s FES-I scores after WBVT, either.

In our study, WBVT did not moderate the severity of fall anxiety in women as measured by the FES-I scale. Pollock et al. [17] did not find changes in the FES-I scores of older women and men after WBVT, either. This similarity of results may be due to insufficient length of WBVT training in both studies. It is likely that WBVT would have a more distinct effect on subjects’ fear of falling if enhanced by psychological support, as reported by Antony G. et al. [25]. Research shows that elderly persons who have suffered a fall limit physical activity not to fall again; as a result, they become more fragile and more prone to another fall [24].

### 4.3. Serum IL-6 Concentrations

Older adults have greater concentrations of pro-inflammatory factors, such as IL-6, compared with younger people [26,27,28]. According to Puzianowska-Kuznicka et al. [29], elevated levels of serum IL-6 are characteristics of older adults and occur regardless of whether they have conditions that might explain them. There is evidence [26,27] to suggest that increased concentration of IL-6 is associated with a secondary autoimmune response to chronic inflammation processes that develop with ageing. Leng et al. [30] with 1106 older women confirmed a relationship between a frailty syndrome and an elevated IL-6 level.

Clinical studies with subjects of different ages [31,32,33,34,35] (including seniors [30,32,33]) show that regular physical activity can reduce the serum concentration of IL-6 and attenuate chronic inflammation. In the study by Broadbent et al. [36], vibrations (40 Hz and 2 mm) applied to the large muscle groups of the lower limbs lowered IL-6 concentrations in 24 relatively young recreational male runners (aged 18–45 years; 33 ± 8 years) [36].

Different results were reported by Hazzel et al. [37]. In their study, young, healthy men (25 ± 3.5 years) performed two vibration training sessions spaced 4 weeks apart. Each session consisted of 30 min of exercises engaging the upper and lower body muscles (press-ups, squats, leg lunges, and backbends). One of the sessions concluded with WBVT (45 Hz, 2 mm). The authors did not find the serum IL-6 concentrations measured after 24 h to be statistically significantly different (*p* > 0.05) between the exercise-and-WBVT session and the exercise-only session [37].

In our study, the serum IL-6 concentrations of participants in the EG measured at week 12 were not statistically significantly different from those obtained at baseline, nor were they statistically significantly correlated with the results of the functional tests (TUG, 6MWT, 30sCST). To our best knowledge, there is only one clinical trial evaluating the effect of WBVT on the serum IL-6 concentration in healthy seniors. Its authors, Cristi et al. [18], had 36 women at a mean age of 81.1 ± 1.2 years perform 3 WBVT sessions per week (30–45 Hz, 2 mm) for 9 weeks (27 sessions in total). The duration of vibrations, separated by 3 min intervals, was progressively increased from 30 to 60 s and the number of sessions from 5 to 11. Similar to our study, Cristi et al. did not find statistically significant changes in IL-6 concentrations measured at week 9 (*p* > 0.05) [18].

Simão et al. [38] suggested the ability of WBVT to lower blood concentrations of pro-inflammatory mediators in older adults, but their study involved 32 subjects aged 65+ with knee osteoarthritis (2–4 points on the Kellgren–Lawrence classification). None of our patients or those studied by Cristi et al. [18] presented with conditions. Simão et al. [38] randomized their patients into three groups: a WBVT group (35–40 Hz, 4 mm) exercising three times a week; a physical exercise group performing squats three times a week; and a control group whose physical activity was limited to daily living routines. The serum concentrations of sTNFR1 and sTNFR2 (soluble tumor necrosis factor receptors) measured in the WBVT group after 12 weeks of exercise were statistically significantly lower (*p* = 0.01) than in controls, but significant differences between the squats group and the control group and between the WBVT group and the squats group were not determined (*p* > 0.05) [38].

The results of the cited studies conducted with relatively young adults and seniors are conflicting and fail to satisfactorily explain the effect of whole-body vibration training on the concentrations of pro-inflammatory factors, including IL-6. More clinical research is, therefore, needed to determine whether and to what extent WBVT influences the factors’ concentrations in seniors and people of other ages.

### 4.4. Clinical Recommendations for Applying WBVT to Women Aged 60+

Our and other authors’ findings [14,15,16,18,38] confirm that WBVT can improve dynamic balance, the strength of the lower limb muscles, and exercise tolerance in older adults. Studies in this field are still few, but the results of most of them are consistent enough to indicate WBVT protocols that can best protect seniors from intrinsic fall risk factors.

The authors of the cited studies applied vibration training to subjects standing on a force plate in a semi-squat position (120–130°) or deep-squat position (90°) [14,15,16,18,38], whichever was more comfortable for them. Some authors also had them perform dynamic exercises, such as squats or leg lunges, while on the plate [14,15]. The frequency of vibrations ranged from 10 to 50 Hz [14,15,16,18,38], and the amplitude from 1 to 5 mm [14,15,16,18,38]; amplitudes of 8 mm [17] or 12 mm [16] were used less frequently. Most authors progressively increased vibration frequency [14,15,16,18,38] and amplitude [14,15,16,18,38] from session to session.

The number of cycles making up an exercise session was increased over the training period from 1 to 5 [14,15,17]. In most cases, a cycle consisted of vibrations applied for 30 to 60 s [14,15,16,18,38] and a 30 or 60-s interval [12,17]. The authors of one study used a 3-min interval [18], and in two studies, intervals were reduced from session to session, from 60 to 5 s [14,15].

The subjects in the cited studies performed WBVT sessions 3 times per week [14,15,16,18,38] for 8 [17],9 [12,16], 12 [16], or 24 weeks [14,15].

Whole-body vibration training using 20 Hz frequency and 2 mm amplitude and applied twice a week for 12 weeks can improve functional gait efficiency, dynamic balance, functional strength of the lower body muscles, and exercise tolerance in women aged 60+ at increased risk of falling. An exercise session should have five cycles, each consisting of 1-min vibrations separated by a 1-min interval. This recommendation is only based the results of our study and its WBVT protocol, so it may not apply to all women at risk of falls. More clinical research comparing our WBVT protocol with other WBTV methodologies is needed to validate its effectiveness.

### 4.5. Novelty, Limitations, and Advantages of the Study

The study is novel in being the first randomized clinical trial that assessed WBVT-induced changes in serum IL-6 concentrations and exercise tolerance in women aged 60+ at increased risk of falls and compared them with non-exercising controls.

The study was conducted in conformity with quality clinical research standards. The patients were randomly allocated to groups, and all exercise sessions were conducted using the same force plate, in the same room, and under the same ambient conditions.

The limitations of the study included a small group size and a lack of a placebo group due to the non-existence of sham WBVT. Also, neither the participants nor the medical personnel in charge of exercise sessions were blinded. The long-term effects of the intervention were not evaluated, either. Therefore, the study’s results should be interpreted with some caution and validated by further randomized clinical trials.

## 5. Conclusions

WBVT improves functional gait efficiency, dynamic balance, exercise tolerance, and functional strength of the lower body muscles in women aged 60+ at risk of falls, but it does not reduce the intensity of their fear of falling or serum IL-6 concentrations. No associations were found between the serum IL-6 concentrations of women aged 60+ at risk of falls and functional gait efficiency, dynamic balance, exercise tolerance, and functional strength of the lower body muscles.

## Figures and Tables

**Table 1:** Variables characterizing patients in the experimental and control groups pre-intervention (n = 42).

Variable	Experimental Group (n = 22)	Control Group (n =20)	*p*
* Age (years):			
Mean ± SD	69.00 ± 6.74	70.69 ± 6.96	0.938
Median (lower quartile—upper quartile)	67.00 (66.00–71.00)	70.00 (67.00–75.00)	
* BMI (kg/m^2^)			
Mean ± SD	28.46 ± 4.57	22.18 ± 12.31	
Median (lower quartile—upper quartile)	28.12 (25.30–32.05)	24.98 (19.56–29.98)	0.092
** Place of residence (no. of women, (%))			
Long-term care facility	9 (40.91%)	7 (35%)	
Own home	13 (59.09%)	13 (65%)	0.211
* Performance in daily activities (score)^1^			
Mean ± SD	98.86 ± 3.43	97.81 ± 4.07	
Median (lower quartile—upper quartile)	100.00 (100.00–100.00)	100 (95–100)	0.103
* Cognitive function (score) ^2^			
Mean ± SD	29.40 ± 1.22	29.12 ± 1.15	
Median (lower quartile—upper quartile)	30.00 (30.00–30.00)	30.00 (28.00–30.00)	0.915
* Intensity of fear of falling (FES-I) (score) ^3^			
Mean ± SD	21.41 ± 5.45	24.56 ± 6.81	
Median (lower quartile—upper quartile)	20.00 (18.00–22.00)	24.00 (20.00–26.00)	0.219
* Functional gait efficiency and dynamic balance (TUG) (s) ^4^			
Mean ± SD	8.62 ± 2.14	10.22 ± 4.49	
Median (lower quartile—upper quartile)	8.44 (6.90–10.08)	9.00 (8.02–9.79)	0.350
* Exercise tolerance (6MWT) (m) ^5^			
Mean ± SD	307.48 ± 74.30	288.19 ± 105.78	
Median (lower quartile—upper quartile)	295.00 (260.00–360.00)	279.00 (220.00–325.00)	0.367
* Functional strength of the lower body muscles (30SCST) (no. of repetitions) ^6^			
Mean ± SD	12.59 ± 3.68	11.31 ± 3.34	
Median (lower quartile—upper quartile)	12.50 (10.00–15.00)	11 (9.00–14.00)	0.319
* Serum concentration of interleukin-6 (pg/mL)			
Mean ± SD	5.31 ± 6.70	4.79 ± 3.38	
Median (lower quartile—upper quartile)	3.31 (2.73–4.94)	3.31 (3.02–5.37)	0.832

SD—standard deviation; BMI—body mass index; ^1^ Barthel Scale; ^2^ Mini Mental Test; ^3^ FES-I—Falls Efficacy Scale International; ^4^ TUG—Timed Up and Go Test; ^5^ Exercise tolerance (6MWT)—Six Minute Walk Test; ^6^ 30SCST—30-Second Chair Stand Test; * Mann–Whitney *U* test; ** maximum likelihood Chi-square test. All tests showed that none of the variables significantly differentiated patients in the experimental group and the control group (*p* > 0.05).

**Table 2:** Between-group comparison of the intervention’s clinical effects (n = 42).

Percentage Change from Baseline	Experimental Group (n = 22)	Control Group (n = 20)	* Level of Significance
	Mean ± SD Median (Lower Quartile—Upper Quartile)		
%TUG (%) ^1^	−17 ± 29 −11 (−3–28)	7 ± 12 7 (11–−3)	0.009
%6MWT (%) ^2^	50 ± 44 48 (10–75)	9 ± 28 4 (−6–11)	0.001
%30SCST (%) ^3^	19 ± 33 13 (−9–36)	−5 ± 15 0 (−17–9)	0.027
%FES-I (%) ^4^	5 ± 17 0 (17–−5)	2 ± 21 −2 (11–−6)	0.655
^1^ %IL-6 (%) ^5^	−2 ± 34 −4 (−15–14)	39 ± 108 8 (3–12)	0.377

* Mann–Whitney *U* test; SD—standard deviation; ^1^ %TUG—percentage change in the time for completing the Timed Up and Go Test; ^2^ %6MWT—percentage change in the distance walked during the Six Minute Walk Test; ^3^ %30SCST—percentage change in the number of repetitions in the 30-s Chair Stand Test; ^4^ %FES-I—percentage change in the intensity of fear of falling measured on the Falls Efficacy Scale International, ^5^ %IL-6—percentage change in serum IL-6 concentration.

**Table 3:** Pre-intervention correlations between IL-6 concentrations and the results of functional tests (n = 42).

Variable	No. of Participants	Correlation Coefficient	* Level of Significance
**Pre-Intervention**			
**Experimental group**			
^1^ IL-6 (pg/mL): ^2^ TUG (s)	22	R = 0.160	*p* = 0.514
^1^ IL-6 (pg/mL): ^3^ 6MWT (m)	22	R = −0.277	*p* = 0.251
^1^ IL-6 (pg/mL): ^4^ 30SCST (no. of repetitions)	22	R = 0.446	*p* = 0.055
**Control group**			
^1^ IL-6 (pg/mL): ^2^ TUG (s)	20	R = 0.310	*p* = 0.462
^1^ IL-6 (pg/mL): ^3^ 6MWT (m)	20	R = −0.169	*p* = 0.695
^1^ IL-6 (pg/mL): ^4^ 30SCST (no. of repetitions)	20	R = 0.072	*p* = 0.872

* Spearman’s correlation of ranks; ^1^ IL-6—interleukin-6; ^2^ TUG—the Timed Up and Go test; ^3^ 6MWT—the Six Minute Walk Test; ^4^ 30SCST—the 30-s Chair Stand Test.

**Table 4:** Post-intervention correlations between IL-6 concentrations and the results of functional tests (n = 42).

Variable	No. of Participants	Correlation Coefficient	* Level of Significance
**Post-Intervention**			
**Experimental group**			
^1^ IL-6 (pg/mL): ^2^ TUG [s]	22	R = 0.227	*p* = 0.349
^1^ IL-6 (pg/mL): ^3^ 6MWT [m]	22	R = −0.050	*p* = 0.838
^1^ IL-6 (pg/mL): ^4^ 30SCST [no. of repetitions]	22	R = 0.416	*p* = 0.076
**Control group**			
^1^ IL-6 [pg/mL]: ^2^ TUG [s]	20	R = 0.638	*p* = 0.098
^1^ IL-6 [pg/mL]: ^3^ 6MWT [m]	20	R = 0.262	*p* = 0.536
^1^ IL-6 [pg/mL]: ^4^ 30SCST [no. of repetitions]	20	R = 0.287	*p* = 0.487

* Spearman’s correlation of ranks; ^1^ IL-6—interleukin-6; ^2^ TUG—the Timed Up and Go test; ^3^ 6MWT—the Six Minute Walk Test; ^4^ 30sCST—the 30-s Chair Stand Test.

## Data Availability

The data presented in this study can be obtained by contacting the corresponding author.
